# Implementation research for taking tobacco control policies to scale in India: a realist evaluation study protocol

**DOI:** 10.1136/bmjopen-2021-050859

**Published:** 2021-05-18

**Authors:** Pragati Bhaskar Hebbar, Vivek Dsouza, Upendra Bhojani, Onno CP van Schayck, Giridhara R Babu, Gera Nagelhout

**Affiliations:** 1 Institute of Public Health Bengaluru, Bangalore, Karnataka, India; 2 Department of Family Medicine, Maastricht University (CAPHRI), Maastricht, Netherlands; 3 Epidemiology, IIPH-H, Bangalore campus, Associate Professor, Public Health Foundation of India, IIPH-H, Bangalore, Karnataka, India; 4 Department of Health Promotion, Maastricht University (CAPHRI), Maastricht, The Netherlands; 5 IVO Research Institute, Koningin Julianaplein, The Hague, Netherlands

**Keywords:** health policy, public health, protocols & guidelines

## Abstract

**Introduction:**

There are ongoing policies and programs to reduce tobacco use and minimise the associated health burden in India. However, there are several challenges in practice leading to different outcomes across Indian states. Inadequate understanding of how national tobacco control policies achieve their results under varied circumstances obstruct the implementation and scaling up of effective strategies. This study is a realist evaluation using largely qualitative methods to understand the implementation process of India’s tobacco control policies. It will do so by evaluating India’s Cigarettes and Other Tobacco Products Act (COTPA) and the National Tobacco Control Program (NTCP). The study aims to examine how, why, for whom and under which circumstances COTPA and NTCP are implemented in India.

**Methods and analysis:**

A realist synthesis on implementation of tobacco control policies in low-income and middle-income countries is conducted. This is followed by qualitative data collection and analysis in three Indian states selected based on data from two rounds of the Global Adult Tobacco Survey. The study comprises of three steps (1): development of initial programme theories, (2) testing and refinement of initial programme theories and (3) testing and validation of refined programme theories. We will interview policy-makers, programme managers and implementers to identify facilitators and barriers of implementation. The purpose is to identify context-specific evidence-based strategies to gain insights into the implementation process of COTPA and NTCP. Further we aim to contribute to tobacco control research by establishing communities of practice to engage with cross-cutting issues.

**Ethics and dissemination:**

The Institutional Ethics Committee, at the Institute of Public Health (Bengaluru), has approved the protocol. Written informed consent forms will be obtained from all the participants. Dissemination has been planned for researchers, policy-makers and implementers as well as the public through peer-reviewed publications, conference presentation, webinars and social media updates.

**PROSPERO registration number:**

CRD42020191541.

**Strengths and limitations of the study**

- Realist synthesis to build on the published literature from diverse disciplines to theorise about implementation of tobacco control policies in low-income and middle-income countries.
- Realist evaluation on how implementation and scaling up of implementation of tobacco control law works in three diverse subnational contexts within India.
- Regional consultations and communities of practice to test and refine theories.
- Secondary data of enforcement and monitoring of tobacco control policies have challenges of limited data reliability, data segregation and comparability over time.
- Primary data collection of interviews and observations might be impacted by COVID-19-related restrictions.

## Introduction

Tobacco use is one of the major public health threats killing approximately 8 million people globally and over 1.3 million adults in India each year.^1^ According to the 2016–2017 Global Adult Tobacco Survey (GATS), 28.6% (roughly 267 million) of the adult population uses tobacco in India. In 2011, 14.6% of adolescents (over 35 million people) were current users of varied forms of tobacco products.^2^ In the same year, the economic burden of tobacco-related diseases in India was INR 1 04 500 crores (US$ 22.4 billion), which was 1.16% of the country’s gross domestic product.^3^

Prior to the WHO Framework Convention on Tobacco Control (FCTC) ratification in 2004, India passed a comprehensive tobacco control law—the Cigarettes and Other Tobacco Products Act (COTPA), 2003 to prohibit (1) smoking in public places, (2) direct and indirect advertisements of tobacco products, (3) sale of tobacco products to minors and within 100 yards of any educational institutions and (4) sale without specified graphic health warning labels.^4^ To support the implementation of this law, the Government of India launched the National Tobacco Control Program (NTCP) in the year 2007–2008 to (1) create awareness regarding the harmful effects of tobacco consumption, (2) reduce the production and supply of tobacco products, (3) ensure effective implementation of the provisions under COTPA 2003, (4) help people quit tobacco use and (5) facilitate implementation strategies for prevention and control of tobacco advocated by the WHO FCTC.^5^ Despite a national policy and programme, the outcomes in Indian states is varied. Existing social inequities are compounded with contextual factors such as the differences in political and administrative structures, organisational and individual capacities, and the political economy of tobacco production and consumption within the various states.^6^

### Complex policies and their implementation

Policy implementation is complex because of a number of interacting actors involved and the environments in which they are designed and implemented (contexts).^7 8^ Several conceptual and theoretical frameworks on understanding policy and its implementation exist across domains such as political science and public administration.^9 10^ For instance, Laswell’s linear model outlines five stages of public policy processes—agenda setting, formulation, adoption, implementation and evaluation, where each stage has its own dynamics and presents several challenges and opportunities.^11^ In the field of public health, Walt and Gilson developed the policy triangle framework to analyse the linkages between the policy contexts, policy processes and policy contents, and its influence on the health outcomes while bringing into focus the important role of state and non-state actors.^12^ Despite the existing frameworks and literature on macrolevel policy analysis, there is a dearth of research and theorisation on the implementation process, especially in low-income and middle-income countries (LMICs).

### Realist evaluation approach

The theory-driven inquiry is considered promising in its attempt to evaluate complex phenomena because it examines the causal mechanisms by considering the contexts within which the intervention is embedded that facilitates a particular kind of change.^13–15^ Realist evaluation, a form of theory-driven inquiry, is increasingly used to assess health policies and programmes, to explain what works for whom and under what circumstances instead of simply ‘does it work or not?’^16^ The goal of realist evaluation is to make sense of conditions in which interventions work (or not) by generating plausible mechanisms. In tobacco control policy implementation, some of these potential conditions are geographic, demographic, economic, religious and cultural factors rooted in complex sociopolitical systems that drive or hinder implementation of policies. The study aims to explain how certain facilitators and barriers shape implementation of COTPA and NTCP in India, which can be used to inform policy-makers about best practices. The current research constitutes a doctoral study that is part of a broader enquiry that aims to inform policy and practice through a Department of Biotechnology/Wellcome Trust early career fellowship grant. The broad research question that is addressed in this study is: How were tobacco control policies implemented in India between 2003 and 2018? This is subdivided into three questions that will guide the study.

- Which states performed better (implementation and outcomes), and for whom (which populations) was it better?
- How do Indian states differ with respect to tobacco control outcomes?
- Which factors facilitated successes in implementation in some settings? How were these achievements sustained? And what were the barriers?

## Methods and analysis

**Figure 1:**
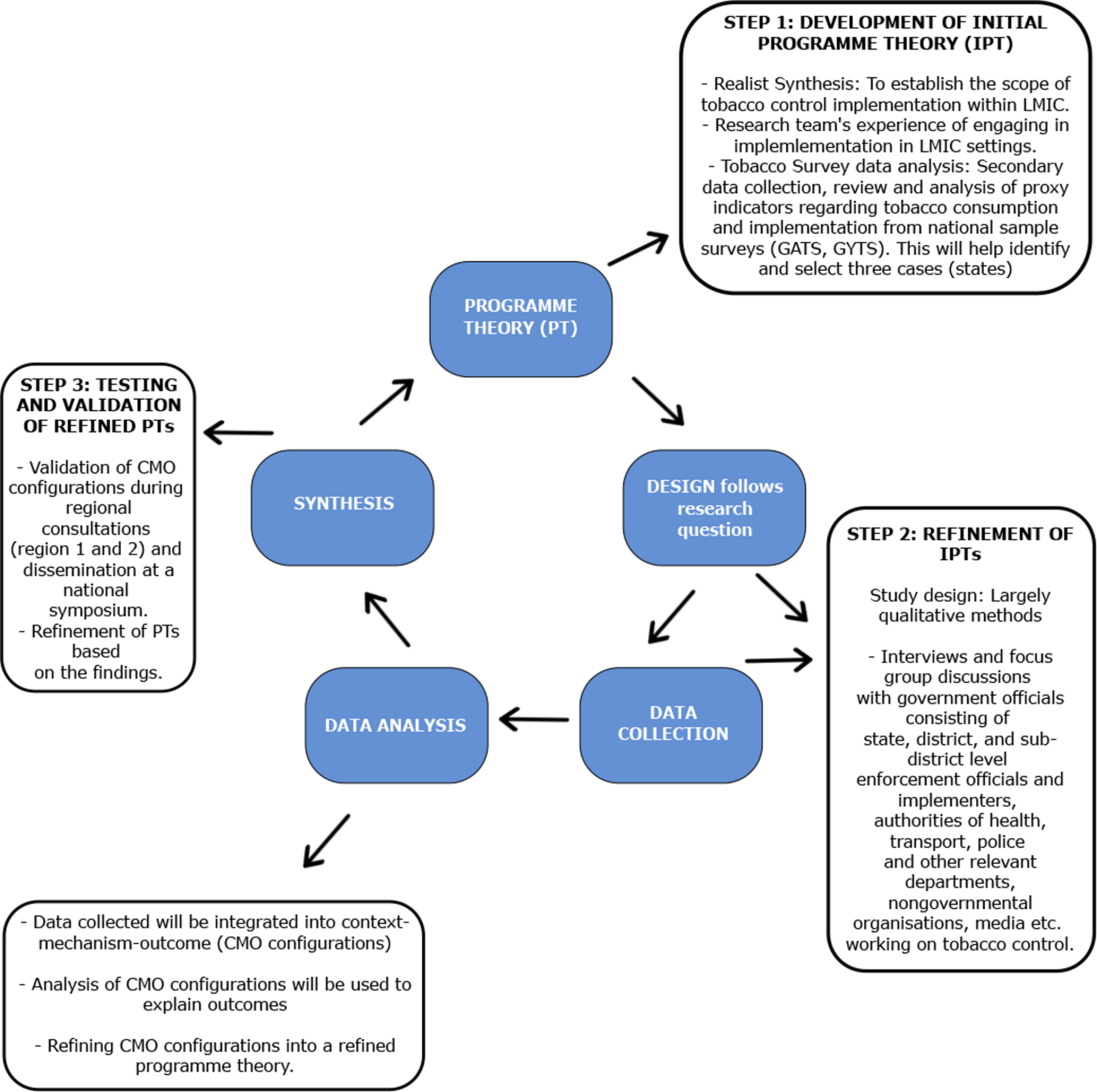
The overall study design which includes the realist synthesis followed by a realist evaluation to develop, test and refine program theories explaining the implementation of tobacco control policies.

The study is a realist evaluation using an implementation research approach to examine and explain the facilitators and barriers to implementing COTPA and NTCP in India. Realist evaluation is method neutral and increasingly used to evaluate complex interventions. This approach will be used to develop, test, validate and refine programme theories (also known as middle-range theories) that explain how the contexts (C) in which COTPA is implemented influences a set of mechanisms (M) resulting in particular kinds of outcomes (O). These C–M–O configurations will help understand how COTPA and NTCP work in different settings to produce different outcomes. The study uses the Realist And Meta-narrative Evidence Syntheses: Evolving Standards II reporting standards for realist evaluation to report the empirical results. The overall study design is depicted in figure 1.

### Step 1: Development of initial program theories (IPTs)

For developing the IPTs, a realist synthesis is undertaken in the phase 1 of the study followed by a realist evaluation in the phase 2. The purpose of the synthesis is to understand the available literature regarding the facilitators and barriers to implementation of tobacco control policies in LMICs. Within the LMIC group the social and political contexts vary widely. This synthesis aims to capture the diversity of the contexts and mechanisms that have worked or failed to generate desirable outcomes in such settings. Databases to be used to find literature include EBSCOHost and Web of Science. Studies from LMICs that describe and elaborate on how and why tobacco control policies are implemented will be considered. Articles are included if (a) published in English and peer-reviewed, (b) it describes the facilitators and barriers to tobacco control policy implementation and (c) discusses mechanisms used in implementing tobacco control policies.

Two reviewers independently screen the abstracts and titles, following which an assessment of quality and rigour is performed. The selected papers are then independently reviewed and coded using common definitions. An initial framework was developed by the research team based on their field experiences and understanding of the existing literature. Based on this initial framework, some codes were derived deductively while there was addition to the list inductively based on the data. Due to the study’s qualitative nature, multiple reflexive intercoder discussions are planned after reviewing full texts to minimise the risk of bias during the study. A detailed realist synthesis protocol has been published earlier in the PROSPERO database.^17^ The study will also be guided by the implementation experiences of the principal investigator and that of the research team that comprises members from LMICs and high income countries coming from diverse backgrounds, which will add a layer of richness to the synthesis process. This would conclude the phase one of the study which comprises of the realist synthesis.

#### Study setting

Following the realist synthesis for the realist evaluation Indian states and union territories were ranked by examining proxy indicators of tobacco control policy implementation and placed into three broad categories: (1) better-performing states that show an improvement in the implementation between 2003 and 2018, (2) average performing states that offer no significant change in the implementation between 2003 and 2018 and (3) poor-performing states where the implementation has worsened between 2003 and 2018. For this exercise, data from the GATS India rounds 1 (2009–2010) and 2 (2016–2017) were assessed. A shortlist of three states in each category was developed and shared with some actors (researchers, policy advocates and implementers/decision-makers). One state was selected from each of the three categories by the actors and the research team for detailed case studies. The three Indian states selected were Kerala, West Bengal and Arunachal Pradesh each with widely varying contextual factors. At the end of this phase, the detailed IPTs will help identify and explore C–M–O configurations of COTPA and NTCP.

### Step 2: Testing and refinement of IPTs

The study will employ predominantly qualitative methods with some quantitative data to refine the IPTs by exploring the C–M–O of COTPA and NTCP in the shortlisted three states. It will consist of:

1. In-depth interviews and focus-group discussions (see sample size and strategy).
2. Review and analysis of implementation documents and data based on the enforcement of COTPA sections from government websites and national crime records bureau portal, review mechanism meetings and COTPA awareness activities to understand and build the case studies.
3. Participant observation during meetings, and site visits, field notes and reflections of the researchers regarding implementation of COTPA–NTCP.

#### Sample size and strategy

Interviews and focus group discussions with state and district level stakeholders (about 15–20 in-depth interviews in each of the three states to capture implementers perspectives and at least one focus group discussion per state) will be conducted to understand the challenges and opportunities in implementing tobacco control policies. The stakeholders are purposively selected and range from state and district level programme staff, bureaucrats, politicians, officers from police, education, finance, transport, municipalities, gram panchayat (local self-government bodies), tourism, non-governmental organisations working on tobacco control and members of the media. An index list will be drawn based on stakeholder mapping for each of the three states and later snowballed to reach out to the relevant stakeholders from these index participants. The information sheet and consent will be sought from stakeholders and an interview guide would guide the data collection.

##### Inclusion criteria for stakeholder interviews and focus group discussions

- Participants must be designated decision-makers/managers or implementers.
- Participants must have worked in the area of tobacco control within the selected states.

#### Data collection, management and analysis

The interviews will be telephonic due to the COVID-19 pandemic and if circumstances permit, they will be conducted in-person, using a semistructured interview guide to elicit the facilitators and barriers to implementing COTPA and NTCP. The probes include the thematic of education, enforcement, intersectoral coordination based on the initial framework developed by the research team. The guide will be adapted to different stakeholder groups (based on preliminary research on their background, role in the implementation process and experience), tested for relevance and refined. The research team member will contact the stakeholders by email and phone explaining the goals and objectives of the study and secure appointments. The team member will also explain the informed consent form and send a copy of it via email and the returned signed copies will be securely stored. The interviews will range between 45 and 60 min and be conducted at a preferred time to minimise stakeholders' inconvenience. As a form of member checking, we will conduct at least one focus group discussion in online or face-to-face format within stakeholder groups/departments with some individuals who had participated in in-depth interviews in each of the three states to understand their knowledge and perception regarding COTPA and NTCP.

Data collected from the interviews and focus groups will be audio-recorded and transcribed in verbatim. The audio recording, transcripts and detailed notes taken during each interview will be saved in a secure repository. Internal team guides are developed with clear documentation flow and procedures including file naming for appropriate data management. Analysis of the interview transcripts will be cross checked by two researchers to check the quality and accuracy and identify any interpretation errors. The qualitative data will be organised in data management software —NVIVO software V.12. In NVIVO, the transcripts will be coded into broad themes relevant to the study. Based on a preliminary coding and analysis, the lead researcher and the research team will identify common themes with the help of thematic analysis that will outline mechanisms that are facilitators or barriers to implementing COTPA in India. The analysis is iterative and guided by the realist approach taking into consideration the complexity and contextual specificity in tobacco control policy implementation in India. Interviews from the three states will be compared and contrasted to enable us to identify similarities, differences and gaps in tobacco control policy implementation in different contexts. Regular reflexive interactions are planned with the lead researchers’ mentor and supervisor from the institute for identifying any issues in the conduct of the study and recalibrating when required. This will allow us to sharpen the understanding of tobacco control policy barriers and facilitators. The output of the data analysis will help in refining our IPTs. The resulting theories will be then validated.

### Step 3: Validation of refined PTs

The refined programme theories will be validated during two regional consultations with stakeholders in India and maybe further refined based on the inputs from the consultations. Dissemination of the validated PTs is planned at a final national symposium to meet the policy objective of the fellowship. The regional consultations will be used to validate preliminary findings and programme theories with stakeholders for feedback to examine whether the analysis is in line with the knowledge, perceptions and beliefs of the stakeholders. Following these regional consultations, we envisage developing a community of practice of the participants of the regional consultation. The aim of this community of practice is to provide a platform to share learnings, challenges as well as best practices across departments and different Indian states. The national symposium will facilitate the development of a stakeholders’ manual which will incorporate a simplified version of the refined programme theories—their relevant C–M–O configurations and best practices to scale implementation of COTPA and NTCP in India.

## Ethics and dissemination

The Institutional Ethics Committee, at the Institute of Public Health (Bengaluru), has approved the protocol in two parts. Phase 1 of the study (based on secondary data) was exempted from review and Phase 2 of the study (largely qualitative) submitted on 26 March 2020 was approved on 28 April 2020 (ref: IEC-FR/01/2020). The team will adhere to the ethical considerations during the conduct of the research as proposed in the submission to the institutional ethics committee and provide them an annual update. The findings of the study will be disseminated using academic and non-academic channels for the scientific community, government and non-government organisations, and the public. This will facilitate dialogue and encourage the uptake and integration of evidence into practice. For the scientific community, the intended research outputs are to publish several journal articles: the realist synthesis of facilitators and barriers of implementation of tobacco control policies in LMICs, the variation in the status of COTPA implementation across Indian states, theories explaining the implementation of COTPA in three Indian states and the refined theories on COTPA implementation in India. These will be published as open access journal articles in peer-reviewed journals and as an open access article-based PhD thesis. The findings will also be presented during seminars and journal clubs organised internally at the Institute of Public Health, Bengaluru, and at national and international public health conferences and webinars.

For national and subnational implementers, an implementers’ manual/toolkit comprising of site-specific knowledge, experience and implementer-wisdom on implementation strategies is planned. Data from the findings and analysis during the study will also be translated into technical reports and policy briefs to update the project progress to its partners and funders. For the public, we aim to develop communication materials that translate scientific information into easily understandable resources on tobacco control implementation. This includes social media posts on different platforms (Facebook and Twitter), quarterly updates disseminated via the Institute of Public Health Bengaluru’s newsletter: Institute of Public Health Connect and issuing relevant press releases and newspaper columns.

We organise bimonthly webinars on tobacco control implementation (called ‘Inside Implementation’ to bring together policy-makers, implementers, the public health community and those working in tobacco control and health to critically engage in dialogue on the issue of tobacco use). Consultations (regional as well as national) to disseminate the interim and final reports on the programme theories of implementation of COTPA and NTCP. And an online and offline ‘communities of practice’ mode of engagement that will bring together state and district-level implementers, policy-makers and diverse stakeholders to work on cross-cutting issues of tobacco control and share best practices in strengthening the implementation process of tobacco control in India.

### Limitations of the study

Some of the limitations envisaged include the unavailability, inconsistency, low quality and inaccuracy of state-level enforcement data. Information from different implementing agencies comprises of different data sets, varying figures, different timelines of data collection, addition of new states and union territories based on the political history of India, and the unavailability of disaggregate data (COTPA violations of Sec 4, 5, 6a and 6b and 7) necessary for the study. Furthermore, the ongoing COVID-19 pandemic has mandated a change in data collection techniques from in-person interviews to an online (telephonic or video call based) format which might impact the assessment of the non-verbal communication of the stakeholders during interviews. Researchers are mindful of this and whenever possible will try to conduct video based or in-person interviews. A final limitation that we want to mention is that the findings from the three Indian states would not be generalisable as it will capture specific data and diversity in terms of implementation outcomes, geography and sociopolitical contexts of the three selected states. Hence, they would provide a starting point for enquiries in similar settings and have to be interpreted accordingly.

### Patient and public involvement

This study protocol describes a realist evaluation study during which participants ranging from various government departments as well as non-government organisations will be involved. The engagement with these participants will be for understanding the tobacco control landscape in India, data collection, regional consultations for theory refinement and validation. The involvement will be deepened and sustained by developing communities of practice to exchange learnings and coproduce knowledge related to implementation of tobacco control policies in India.

## Supplementary Material

Reviewer comments

Author's manuscript
