# Supplementary material for: Implementation research for taking tobacco control policies to scale in India: a realist evaluation study protocol
Source: BMJ Open. 2021 May 18;11(5):e050859. doi: 10.1136/bmjopen-2021-050859 (PMC8130733; doi:10.1136/bmjopen-2021-050859)
Supplement: Author's manuscript [file bmjopen-2021-050859.draft_revisions.pdf]

BMJ Open

BMJ Open is committed to open peer review. As part of this commitment we make the peer review history of every article we publish publicly available.

When an article is published we post the peer reviewers' comments and the authors' responses online. We also post the versions of the paper that were used during peer review. These are the versions that the peer review comments apply to.

The versions of the paper that follow are the versions that were submitted during the peer review process. They are not the versions of record or the final published versions. They should not be cited or distributed as the published version of this manuscript.

BMJ Open is an open access journal and the full, final, typeset and author-corrected version of record of the manuscript is available on our site with no access controls, subscription charges or pay-per-view fees (http://bmjopen.bmj.com).

If you have any questions on BMJ Open's open peer review process please email info.bmjopen@bmj.com

BMJ Open

Implementation Research for taking Tobacco Control Policies to scale in India – A realist evaluation study protocol

Journal:	*BMJ Open*
Manuscript ID	bmjopen-2021-050859
Article Type:	Protocol
Date Submitted by the Author:	02-Mar-2021
Complete List of Authors:	Hebbar, Pragati; Institute of Public Health Karnataka, Chronic conditions and public policies<br>Dsouza, Vivek; Institute of Public Health Bengaluru<br>Bhojani, Upendra; Institute of Public Health Bengaluru,<br>van Schayck, Onno; Maastricht University, HAG<br>Babu, Giridhara R; Associate Professor, Public Health Foundation of India, IIPH-H,, Epidemiology, IIPH-H, Bangalore campus; Jonathan and Karen Fielding School of Public health, Department of Epidemiology<br>Nagelhout, Gera; Maastricht University, Health Promotion
Keywords:	Health policy < HEALTH SERVICES ADMINISTRATION & MANAGEMENT, Public health < INFECTIOUS DISEASES, Protocols & guidelines < HEALTH SERVICES ADMINISTRATION & MANAGEMENT

SCHOLARONE™
Manuscripts

I, the Submitting Author has the right to grant and does grant on behalf of all authors of the Work (as defined in the below author licence), an exclusive licence and/or a non-exclusive licence for contributions from authors who are: i) UK Crown employees; ii) where BMJ has agreed a CC-BY licence shall apply, and/or iii) in accordance with the terms applicable for US Federal Government officers or employees acting as part of their official duties; on a worldwide, perpetual, irrevocable, royalty-free basis to BMJ Publishing Group Ltd ("BMJ") its licensees and where the relevant Journal is co-owned by BMJ to the co-owners of the Journal, to publish the Work in this journal and any other BMJ products and to exploit all rights, as set out in our licence.

The Submitting Author accepts and understands that any supply made under these terms is made by BMJ to the Submitting Author unless you are acting as an employee on behalf of your employer or a postgraduate student of an affiliated institution which is paying any applicable article publishing charge ("APC") for Open Access articles. Where the Submitting Author wishes to make the Work available on an Open Access basis (and intends to pay the relevant APC), the terms of reuse of such Open Access shall be governed by a Creative Commons licence – details of these licences and which Creative Commons licence will apply to this Work are set out in our licence referred to above.

Other than as permitted in any relevant BMJ Author's Self Archiving Policies, I confirm this Work has not been accepted for publication elsewhere, is not being considered for publication elsewhere and does not duplicate material already published. I confirm all authors consent to publication of this Work and authorise the granting of this licence.

For peer review only

Title page

Title: Implementation Research for taking Tobacco Control Policies to scale in India – A realist evaluation study protocol.

Authors: *Pragati Hebbar[1,4], Vivek Dsouza[1], Upendra Bhojani[1], Onno C P van Schayck[2], Giridhara R Babu[3], Gera E. Nagelhout[4,5]

[1]Institute of Public Health (IPH), 3009, II-A Main, 17th Cross, Krishna Rajendra Rd, Banashankari Stage II, Bengaluru, Karnataka 560070.

[2]Department of Family Medicine, Maastricht University (CAPHRI), P. Debyeplein 1, 6229 HA Maastricht, Netherlands.

[3]Indian Institute of Public Health, Public Health Foundation of India (PHFI), Bangalore, Magadi Rd 1st cross, Next to leprosy hospital, SIHFW premises, Bengaluru, Karnataka 560023

[4]Department of Health Promotion, Maastricht University (CAPHRI), P. Debyeplein 1, 6229 HA Maastricht, the Netherlands.

[5] IVO Research Institute, Koningin Julianaplein 10, 2595 AA The Hague, the Netherlands

***Corresponding Author**: Pragati B Hebbar (pragati@iphindia.org)

For peer review only - http://bmjopen.bmj.com/site/about/guidelines.xhtml

Abstract

Introduction: There are ongoing policies and programs to reduce tobacco use and minimize the associated health burden in India. However, there are several challenges in practice leading to different outcomes across Indian states. Inadequate understanding of how national tobacco control policies achieve their results under varied circumstances obstruct the implementation and scaling-up of effective strategies. This study is a realist evaluation using largely qualitative methods to understand the implementation process of India's tobacco control policies. It will do so by evaluating India's Cigarettes and Other Tobacco Products Act (COTPA) and the National Tobacco Control Program (NTCP). The study aims to examine how, why, for whom, and under which circumstances COTPA and NTCP are implemented in India.

Methods and analysis: A realist synthesis on implementation of tobacco control policies in low- and middle-income countries is conducted. This is followed by qualitative data collection and analysis in three Indian states selected based on data from two rounds of the Global Adult Tobacco Survey. The study comprises of three steps: (1) development of initial program theories, (2) testing and refinement of initial program theories, and (3) testing and validation of refined program theories. We will interview policymakers, program managers, and implementers to identify facilitators and barriers of implementation. The purpose is to identify context-specific evidence-based strategies to gain insights into the implementation process of COTPA and NTCP. Further we aim to contribute to tobacco control research by establishing communities of practice to engage with cross-cutting issues

Ethics and dissemination: The Institutional Ethics Committee, at the Institute of Public Health (Bengaluru) has approved the protocol. Written informed consent forms will be obtained from all the participants. Dissemination has been planned for researchers, policy

For peer review only - http://bmjopen.bmj.com/site/about/guidelines.xhtml

makers and implementers as well as the public through peer reviewed publications, conference presentation, webinars, social media updates.

PROSPERO registration: CRD42020191541

KEYWORDS: Realist evaluation, Tobacco control, Implementation, low and middle-income countries, Public health, India.

Strengths and limitations of the study

- There is a dearth of research and theorization on the implementation process, especially in low middle-income countries (LMICs). This study will generate empirical data and theories to fill in this gap
- The study will add to the existing literature on implementation frameworks by explaining implementation of tobacco control policies through detailed case studies of select Indian states
- Application of realist evaluation to explain the implementation process of a large national policy and program will be a novel addition to the growing body of literature on realist evaluation
- The findings from the three Indian states would not be generalizable as it will capture specific data and diversity in terms of implementation outcomes, geography and socio-political contexts of the three selected states. Hence, they would provide a starting point for enquiries in similar settings and should be interpreted accordingly

For peer review only

For peer review only - http://bmjopen.bmj.com/site/about/guidelines.xhtml

Introduction

Tobacco use is one of the major public health threats killing approximately eight million people globally and over 1.3 million adults in India each year.(1) According to the 2016-2017 Global Adult Tobacco Survey, 28.6% (roughly 267 million) of the adult population uses tobacco in India. In 2011, 14.6% of adolescents (over 35 million people) were current users of varied forms of tobacco products.(2) In the same year, the economic burden of tobacco-related diseases in India was INR. 1,04,500 crores (US$ 22.4 billion) which was 1.16% of the country's GDP.(3)

Prior to the WHO Framework Convention on Tobacco Control (FCTC) ratification in 2004, India passed a comprehensive tobacco control law – the Cigarettes and Other Tobacco Products Act (COTPA), 2003 to prohibit (1) smoking in public places, (2) direct and indirect advertisements of tobacco products, (3) sale of tobacco products to minors and within 100 yards of any educational institutions, and (4) sale without specified graphic health warning labels.(4) To support the implementation of this law, the Government of India launched the National Tobacco Control Program (NTCP) in the year 2007-08 to 1) create awareness regarding the harmful effects of tobacco consumption, 2) reduce the production and supply of tobacco products, 3) ensure effective implementation of the provisions under 'The COTPA 2003, 4) help people quit tobacco use, and 5) facilitate implementation strategies for prevention and control of tobacco advocated by the WHO FCTC.(5) Despite a national policy and program, the outcomes in Indian states is varied. Existing social inequities are compounded with contextual factors such as the differences in political and administrative structures, organizational and individual capacities, and the political economy of tobacco production and consumption within the various states.(6)

Complex policies and their implementation

For peer review only - http://bmjopen.bmj.com/site/about/guidelines.xhtml

Policy implementation is complex because of a number of interacting actors involved and the environments in which they are designed and implemented (contexts).(7)(8) Several conceptual and theoretical frameworks on understanding policy and its implementation exist across domains like political science and public administration.(9)(10) For instance, Laswell's linear model outlines five stages of public policy processes – agenda setting, formulation, adoption, implementation, and evaluation, where each stage has its own dynamics and presents several challenges and opportunities.(11) In the field of public health, Walt and Gilson developed the policy triangle framework to analyze the linkages between the policy contexts, policy processes and policy contents, and its influence on the health outcomes while bringing into focus the important role of state and non-state actors.(12) Despite the existing frameworks and literature on macro-level policy analysis, there is a dearth of research and theorization on the implementation process, especially in low and middle-income countries (LMICs).

Realist evaluation approach

The theory-driven inquiry is considered promising in its attempt to evaluate complex phenomena because it examines the causal mechanisms by considering the contexts within which the intervention is embedded that facilitates a particular kind of change.(13)(14)(15) Realist evaluation, a form of theory-driven inquiry, is increasingly used to assess health policies and programs, to explain *what* works for *whom* and under *what circumstances* instead of simply 'does it work or not?'(16) The goal of realist evaluation is to make sense of *conditions* in which interventions work (or not) by generating plausible mechanisms. In tobacco control policy implementation, some of these potential conditions are geographic, demographic, economic, religious, and cultural factors rooted in complex socio-political systems that drive or hinder implementation of policies. The study aims to explain how

For peer review only

certain facilitators and barriers shape implementation of COTPA and NTCP in India, which can be used to inform policymakers about best practices. The current research constitutes a doctoral study that is part of a broader enquiry that aims to inform policy and practice through a DBT/ Wellcome Trust early career fellowship grant. The broad research question that is addressed in this study is: How were tobacco control policies implemented in India between 2003-2018? This is subdivided into three questions that will guide the study.

a. Which states performed better (implementation and outcomes), and for whom (which populations) was it better?
b. How do Indian states differ with respect to tobacco control outcomes?
c. Which factors facilitated successes in implementation in some settings? How were these achievements sustained? And what were the barriers?

Methods and analysis

The study is a realist evaluation using an implementation research approach to examine and explain the facilitators and barriers to implementing COTPA and NTCP in India. Realist evaluation is method neutral and increasingly used to evaluate complex interventions. This approach will be used to develop, test, validate, and refine program theories (also known as middle-range theories) that explain how the contexts (C) in which COTPA is implemented influences a set of mechanisms (M) resulting in particular kinds of outcomes (O). These C-M-O configurations will help understand how COTPA and NTCP work in different settings to produce different outcomes. The study uses the Realist And Meta-narrative Evidence Syntheses: Evolving Standards (RAMESES) II reporting standards for realist evaluation to report the empirical results. The overall study design is depicted in Figure 1.

Step 1: Development of Initial Program Theories (IPTs)

For peer review only - http://bmjopen.bmj.com/site/about/guidelines.xhtml

For developing the IPTs, a realist synthesis is undertaken in the phase 1 of the study followed by a realist evaluation in the phase 2. The purpose of the synthesis is to understand the available literature regarding the facilitators and barriers to implementation of tobacco control policies in LMICs. Within the LMIC group the social and political contexts vary widely. This synthesis aims to capture the diversity of the contexts and mechanisms that have worked or failed to generate desirable outcomes in such settings. Databases to be used to find literature include EBSCOHost and Web of Science. Studies from LMICs that describe and elaborate on how and why tobacco control policies are implemented will be considered. Articles are included if (a) published in English and peer-reviewed (b) it describes the facilitators and barriers to tobacco control policy implementation, (c) discusses mechanisms used in implementing tobacco control policies.

Two reviewers independently screen the abstracts and titles, following which an assessment of quality and rigor is performed. The selected papers are then independently reviewed and coded using common definitions. An initial framework was developed by the research team based on their field experiences and understanding of the existing literature. Based on this initial framework, some codes were derived deductively while there was addition to the list inductively based on the data. Due to the study's qualitative nature, multiple reflexive inter-coder discussions are planned after reviewing full texts to minimize the risk of bias during the study. A detailed realist synthesis protocol has been published earlier in the PROSPERO database.(17) The study will also be guided by the implementation experiences of the principal investigator (PH) and that of the research team that comprises members from LMICs and high income countries coming from diverse backgrounds, which will add a layer of richness to the synthesis process. This would conclude the phase one of the study which comprises of the realist synthesis

For peer review only - http://bmjopen.bmj.com/site/about/guidelines.xhtml

Study Setting: Following the realist synthesis for the realist evaluation Indian states and union territories were ranked by examining proxy indicators of tobacco control policy implementation and placed into three broad categories: (1) better-performing states that show an improvement in the implementation between 2003 – 2018, (2) average performing states that offer no significant change in the implementation between 2003 – 2018 and (3) poor-performing states where the implementation has worsened between 2003 – 2018. For this exercise, data from the Global Adult Tobacco Survey (GATS) India rounds 1 (2009-2010) and 2 (2016 – 2017) were assessed. A shortlist of three states in each category was developed and shared with some actors (researchers, policy advocates and implementers/ decision-makers). One state was selected from each of the three categories by the actors and the research team for detailed case studies. The three Indian states selected are Kerala, West Bengal and Arunachal Pradesh each with widely varying contextual factors. At the end of this phase, the detailed IPTs will help identify and explore contexts, mechanisms, and outcomes (C-M-O) configurations of COTPA and NTCP.

Step 2: Testing and refinement of IPTs

The study will employ predominantly qualitative methods with some quantitative data to refine the initial program theories (IPTs) by exploring the contexts, mechanisms, and outcomes, of COTPA and NTCP in the shortlisted three states. It will consist of:

1. In-depth interviews and focus-group discussions (See sample size and strategy)
2. Review and analysis of implementation documents and data based on the enforcement of COTPA sections from government websites and national crime records bureau portal, review mechanism meetings, and COTPA awareness activities to understand and build the case studies.
3. Participant observation during meetings, and site visits, field notes and reflections of the researchers regarding implementation of COTPA-NTCP.

For peer review only

For peer review only - http://bmjopen.bmj.com/site/about/guidelines.xhtml

Sample Size and Strategy: Interviews and focus group discussions with state and district level stakeholders (about 15-20 in-depth interviews in each of the three states to capture implementers perspectives and at least one focus group discussion per state) will be conducted to understand the challenges and opportunities in implementing tobacco control policies. The stakeholders are purposively selected and range from state and district level program staff, bureaucrats, politicians, officers from police, education, finance, transport, municipalities, gram panchayat (local self-government bodies), tourism, non-governmental organizations working on tobacco control, members of the media etc. An index list will be drawn based on stakeholder mapping for each of the three states and later snowballed to reach out to the relevant stakeholders from these index participants. The information sheet and consent will be sought from stakeholders and an interview guide would guide the data collection.

Inclusion criteria for stakeholder interviews and focus group discussions:

- Participants must be designated decision makers/ managers or implementers
- Participants must have worked in the area of tobacco control within the selected states.

Data collection, management, and analysis: The interviews will be telephonic due to the COVID-19 pandemic and if circumstances permit, they will be conducted in-person, using a semi-structured interview guide to elicit the facilitators and barriers to implementing COTPA and NTCP. The probes include the thematic of education, enforcement, intersectoral coordination based on the initial framework developed by the research team. The guide will be adapted to different stakeholder groups (based on preliminary research on their background, role in the implementation process and experience), tested for relevance, and refined. The research team member will contact the stakeholders by email and phone

For peer review only - http://bmjopen.bmj.com/site/about/guidelines.xhtml

explaining the goals and objectives of the study and secure appointments. The team member will also explain the informed consent form and send a copy of it via email and the returned signed copies will be securely stored. The interviews will range between 45-60 minutes and be conducted at a preferred time to minimize stakeholders' inconvenience. As a form of member checking, we will conduct at least one focus group discussion in online or face to face format within stakeholder groups/departments with some individuals who had participated in in-depth interviews in each of the three states to understand their knowledge and perception regarding COTPA and NTCP.

Data collected from the interviews and focus groups will be audio-recorded and transcribed in verbatim. The audio recording, transcripts, and detailed notes taken during each interview will be saved in a secure repository. Internal team guides are developed with clear documentation flow and procedures including file naming for data appropriate management. Analysis of the interview transcripts will be cross checked by two researchers to check the quality and accuracy and identify any interpretation errors. The qualitative data will be organized in data management software -- NVIVO software version 12. In NVIVO, the transcripts will be coded into broad themes relevant to the study. Based on a preliminary coding and analysis, the lead researcher and the research team will identify common themes with the help of thematic analysis that will outline mechanisms that are facilitators or barriers to implementing COTPA in India. The analysis is iterative and guided by the realist approach taking into consideration the complexity and contextual specificity in tobacco control policy implementation in India. Interviews from the three states will be compared and contrasted to enable us to identify similarities, differences, and gaps in tobacco control policy implementation in different contexts. Regular reflexive interactions are planned with the lead researchers' mentor and supervisor from the institute for identifying any issues in the conduct of the study and recalibrating when required. This will allow us to sharpen the understanding

For peer review only

For peer review only - http://bmjopen.bmj.com/site/about/guidelines.xhtml

of tobacco control policy barriers and facilitators. The output of the data analysis will help in refining our IPTs. The resulting theories will be then validated.

Step 3: Validation of refined PTs

The refined program theories will be validated during two regional consultations with stakeholders in India and maybe further refined based on the inputs from the consultations. Dissemination of the validated PTs is planned at a final national symposium to meet the policy objective of the fellowship. The regional consultations will be used to validate preliminary findings and program theories with stakeholders for feedback to examine whether the analysis is in line with the knowledge, perceptions, and beliefs of the stakeholders. Following these regional consultations, we envisage developing a community of practice of the participants of the regional consultation. The aim of this community of practice is to provide a platform to share learnings, challenges as well as best practices across departments and different Indian states. The national symposium will facilitate the development of a stakeholders' manual which will incorporate a simplified version of the refined program theories – their relevant C-M-O configurations and best practices to scale implementation of COTPA and NTCP in India.

Ethics and dissemination

The Institutional Ethics Committee, at the Institute of Public Health (Bengaluru) has approved the protocol in two parts. Phase 1 of the study (based on secondary data) was exempted from review, and Phase 2 of the study (largely qualitative) submitted on March 26, 2020, was approved on April 28, 2020 (ref: IEC-FR/01/2020). The team will adhere to the ethical considerations during the conduct of the research as proposed in the submission to the institutional ethics committee and provide them an annual update. The findings of the study

For peer review only - http://bmjopen.bmj.com/site/about/guidelines.xhtml

will be disseminated using academic and non-academic channels for the scientific community, government and non-government organizations, and the public. This will facilitate dialogue and encourage the uptake and integration of evidence into practice. For the scientific community, the intended research outputs are to publish several journal articles: the realist synthesis of facilitators and barriers of implementation of tobacco control policies in LMICs, the variation in the status of COTPA implementation across Indian states, theories explaining the implementation of COTPA in three Indian states, and the refined theories on COTPA implementation in India. These will be published as open access journal articles in peer-reviewed journals and as an open-access article-based PhD thesis. The findings will also be presented during seminars and journal clubs organized internally at the Institute of Public Health, Bengaluru, and at national and international public health conferences and webinars.

For national and sub-national implementers, an implementers' manual/toolkit comprising of site-specific knowledge, experience, and implementer-wisdom on implementation strategies is planned. Data from the findings and analysis during the study will also be translated into technical reports and policy briefs to update the project progress to its partners and funders. For the public, we aim to develop communication materials that translate scientific information into easily understandable resources on tobacco control implementation. This includes social media platforms (Facebook and Twitter), quarterly updates disseminated via the Institute of Public Health Bengaluru's newsletter: IPH connect and issuing relevant press releases and newspaper columns.

We organize bi-monthly webinars on tobacco control implementation (called 'Inside Implementation' to bring together policymakers, implementers, the public health community, and those working in tobacco control and health to critically engage in dialogue on the issue of tobacco use). Consultations (regional as well as national) to disseminate the interim and final reports on the program theories of implementation of COTPA and NTCP. And an online

For peer review only - http://bmjopen.bmj.com/site/about/guidelines.xhtml

and offline 'communities of practice' mode of engagement that will bring together state and district-level implementers, policy makers, and diverse stakeholders to work on cross-cutting issues of tobacco control and share best practices in strengthening the implementation process of tobacco control in India.

Limitations of the study

Some of the limitations envisaged include the unavailability, inconsistency, low quality, and inaccuracy of state-level enforcement data. Information from different implementing agencies comprises of different data sets, varying figures, different timelines of data collection, addition of new states and union territories based on the political history of India, and the unavailability of disaggregate data (COTPA violations of Sec 4, 5, 6a and 6b, and 7) necessary for the study. Furthermore, the ongoing COVID-19 pandemic has mandated a change in data collection techniques from in-person interviews to an online (telephonic or video call based) format which might impact the assessment of the non-verbal communication of the stakeholders during interviews. Researchers are mindful of this and whenever possible will try to conduct video based or in-person interviews. A final limitation that we want to mention is that the findings from the three Indian states would not be generalizable as it will capture specific data and diversity in terms of implementation outcomes, geography and socio-political contexts of the three selected states. Hence, they would provide a starting point for enquiries in similar settings and have to be interpreted accordingly.

Patient and public involvement

This study protocol describes a realist evaluation study during which participants ranging from various government departments as well as non-government organizations will be

For peer review only - http://bmjopen.bmj.com/site/about/guidelines.xhtml

involved. The engagement with these participants will be for understanding the tobacco control landscape in India, data collection, regional consultations for theory refinement and validation. The involvement will be deepened and sustained by developing communities of practice to exchange learnings and co-produce knowledge related to implementation of tobacco control policies in India.

Acknowledgements – The authors would like to thank Dr. Prashanth NS, DBT/ Wellcome Trust India Alliance Intermediate Fellow at the Institute of Public Health, Bengaluru for his support and inputs on realist evaluation.

For peer review only

References:

1. WHO. WHO report on the global tobacco epidemic, 2019: offer help to quit tobacco use: executive summary. World Heal Organ [Internet]. 2019;1–8. Available from: https://www.who.int/teams/health-promotion/tobacco-control/who-report-on-the-global-tobacco-epidemic-2019
2. WHO. India (Ages 13-15) Global Youth Tobacco Survey (GYTS). Fact Sheet. World Heal Organ [Internet]. 2009;63. Available from: http://www.searo.who.int/entity/noncommunicable_diseases/data/ind_gyts_fs_2009.pdf?ua=1
3. John RM,Rout SK,Kumar BR AM. Report on Economic Burdent of Tobacco Related Diseases in India.pdf. 2014. p. 15–8.
4. The G-I. India - COTPA, 2003 - national.pdf. 2003. p. 9.
5. National Tobacco Control Cell, MOHFW G. Operational Guidelines- National Tobacco Control Programme 2015. 2015;1–66. Available from: http://www.who.int/lep/resources/SEAGLP20062.pdf

6. Nazar GP, Chang KCM, Srivastava S, Pearce N, Karan A, Millett C. Impact of India's National Tobacco Control Programme on bidi and cigarette consumption: a difference-in-differences analysis. Tob Control [Internet]. 2020;29(1):103–10. Available from: http://login.ezproxy.ub.unimaas.nl/login?url=https://search.ebscohost.com/login.aspx?direct=true&db=cmedm&AN=30554161&site=ehost-live&scope=site
7. Sterman JD. Learning from evidence in a complex world. Am J Public Health. 2006;96(3):505–14.
8. Westhorp G. Using complexity-consistent theory for evaluating complex systems. Evaluation. 2012;18(4):405–20.
9. Nilsen P. Making sense of implementation theories, models and frameworks. Implement Sci. 2015;10(1):1–13.
10. Peters DH, Adam T, Alonge O, Agyepong IA, Tran N. Implementation research: What it is and how to do it. Br Med J. 2014;48(8):731–6.
11. Sabatier P. Theories of the Policy Process. Theor Policy Process. 2019;4–7.
12. Walt G, Gilson L. Reforming the health sector in developing countries: The central role of policy analysis. Health Policy Plan. 1994;9(4):353–70.
13. Chen HT, Rossi PH. The theory-driven approach to validity. Eval Program Plann. 1987;10(1):95–103.
14. Van Belle SB, Marchal B, Dubourg D, Kegels G. How to develop a theory-driven evaluation design? Lessons learned from an adolescent sexual and reproductive health programme in West Africa. BMC Public Health. 2010;10.
15. Shearn K, Allmark P, Piercy H, Hirst J. Building Realist Program Theory for Large Complex and Messy Interventions. Int J Qual Methods. 2017;16(1):1–11.
16. Pawson R, Tilley N. An Introduction to Scientific Realist Evaluation. In: Evaluation for the 21st Century: A Handbook. 1997. p. 405.

For peer review only

For peer review only - http://bmjopen.bmj.com/site/about/guidelines.xhtml

17. Hebbar Pragati, Dsouza V, Bhojani U, Schayck OC van, Babu GR, Nagelhout GE. PROSPERO registration. 2020.

Authors' contributions – PH was responsible for the funding acquisition and project administration. UB and PH were involved in conceptualization and GN helped in strengthening the methodology. VD and PH were involved in data collection, analysis, writing original drafts. Whereas UB, OvS, GB and GN were involved in supervision, reviewing and editing drafts. All authors read and approved the final manuscript.

Funding statement - The study is supported by the DBT/Wellcome Trust India Alliance Early Career Fellowship awarded to Pragati Hebbar [IA/ CPHE/17/1/503338]

Competing interests – None declared

Patient consent for publication – Not required

List of abbreviations:

COTPA – Cigarettes and Other Tobacco Products Act

CMO – Context – Mechanism - Outcome

DBT – Department of Biotechnology

GATS – Global Adult Tobacco Survey

IPT – Initial program theory

LMIC – Low- and middle-income countries

NTCP – National Tobacco Control Program

PT – Program theory

RAMESES - Realist And Meta-narrative Evidence Syntheses: Evolving Standards

WHO – FCTC – World Health Organization Framework Convention on Tobacco Control

For peer review only

For peer review only - http://bmjopen.bmj.com/site/about/guidelines.xhtml

Figure title and legend:

Figure title - Figure 1: Study design

Figure legend – Figure 1 explains the overall study design which includes the realist synthesis followed by a realist evaluation to develop, test and refine program theories explaining the implementation of tobacco control policies.

For peer review only

STEP 1: DEVELOPMENT OF INITIAL PROGRAMME THEORY (IPT)

- Realist synthesis: To establish the scope of tobacco control implementation within LMIC regions.
- Research team's experiences of engaging in implementation in LMIC settings.
- Global adult tobacco survey (GATS) data analysis: Secondary data collection, review and analysis of proxy indicators of tobacco control implementation to identify and select three cases (states)

STEP 3: TESTING AND VALIDATION OF REFINED PTs

- Validation of CMO configurations during regional consultations (region 1 and 2) and dissemination at a national symposium.
- Refinement of PTs based on the findings.

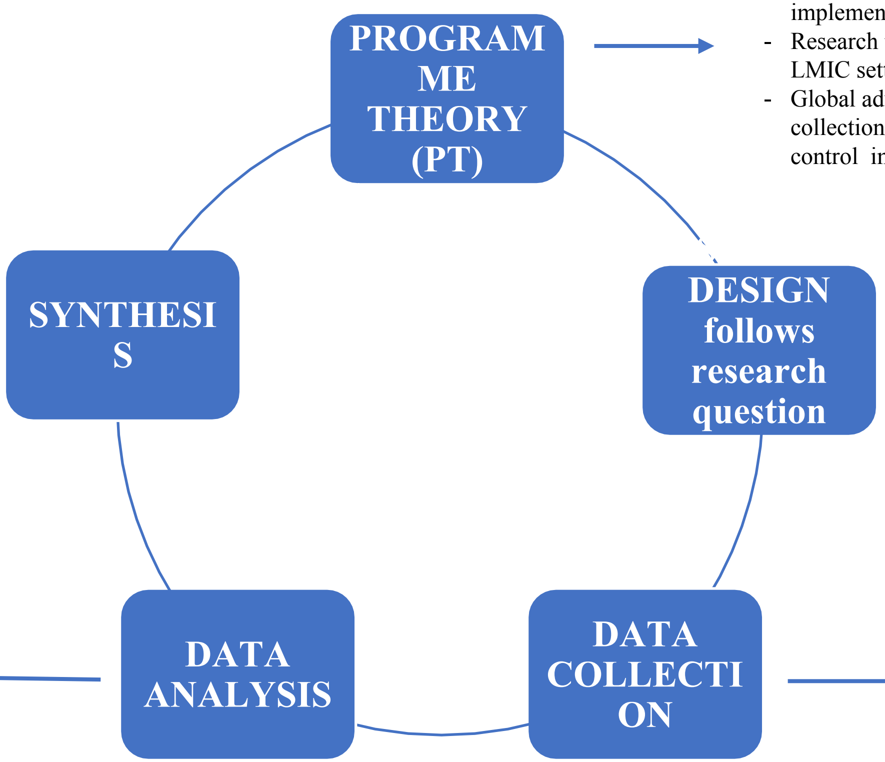

STEP 2: TESTING AND REFINEMENT OF IPTs

Study design: Largely qualitative methods

- Interviews and focus group discussions with government officials consisting of state, district, and sub-district level enforcement officials and implementers, authorities of health, transport, police and other relevant departments, non-governmental organisations, media etc. working on tobacco control.

- Data collected will be integrated into context-mechanism-outcome (CMO) configurations.
- Analysis of CMO configurations will be used to explain outcomes.
- Refining CMO configurations into a refined programme theory.

Jisc Digital Resources > Open Access

Sherpa Juliet

India Alliance

Funder Information

Funder Name	Wellcome Trust/DBT India Alliance (India Alliance) [English]
Funder URLs	https://www.indiaalliance.org/ [English]
Identifiers	FundRef DOI: 10.13039/501100009053<br>ROR ID: https://ror.org/04reqzt68
Country	India
Group Memberships	Europe PMC Funders' Group

Requires Open Access Archiving

Encourages Open Access Publishing

General Open Access Policy

Encourages Open Data Archiving

Record Information

Suggest an update for this record

Useful links

Open access services from Jisc

Guide to managing open access costs

Open access guides by Jisc

Contact

help@jisc.ac.uk

Accessibility

Accessibility Statement

Feedback

☹ 😐 ☺ Give Feedback

Jisc

Digital resources

Maps and spatial data

Learning and research resources

Library support

Open access (OA)

We provide secure, cost-effective access to the UK's richest collection of digital content: giving you access to the latest data and content from leading international publishers and providers.

Find out more at jisc.ac.uk

This work is licensed under the CC BY-NC-ND 4.0
About using our content

BMJ Open

Implementation Research for taking Tobacco Control Policies to scale in India – A realist evaluation study protocol

Journal:	*BMJ Open*
Manuscript ID	bmjopen-2021-050859.R1
Article Type:	Protocol
Date Submitted by the Author:	16-Apr-2021
Complete List of Authors:	Hebbar, Pragati; Institute of Public Health Karnataka, Chronic conditions and public policies<br>Dsouza, Vivek; Institute of Public Health Bengaluru<br>Bhojani, Upendra; Institute of Public Health Bengaluru,<br>van Schayck, Onno; Maastricht University, HAG<br>Babu, Giridhara R; Associate Professor, Public Health Foundation of India, IIPH-H,, Epidemiology, IIPH-H, Bangalore campus; Jonathan and Karen Fielding School of Public health, Department of Epidemiology<br>Nagelhout, Gera; Maastricht University, Health Promotion
**Primary Subject Heading**:	Smoking and tobacco
Secondary Subject Heading:	Health policy, Health services research, Public health, Qualitative research, Global health
Keywords:	Health policy < HEALTH SERVICES ADMINISTRATION & MANAGEMENT, Public health < INFECTIOUS DISEASES, Protocols & guidelines < HEALTH SERVICES ADMINISTRATION & MANAGEMENT

SCHOLARONE™
Manuscripts

I, the Submitting Author has the right to grant and does grant on behalf of all authors of the Work (as defined in the below author licence), an exclusive licence and/or a non-exclusive licence for contributions from authors who are: i) UK Crown employees; ii) where BMJ has agreed a CC-BY licence shall apply, and/or iii) in accordance with the terms applicable for US Federal Government officers or employees acting as part of their official duties; on a worldwide, perpetual, irrevocable, royalty-free basis to BMJ Publishing Group Ltd ("BMJ") its licensees and where the relevant Journal is co-owned by BMJ to the co-owners of the Journal, to publish the Work in this journal and any other BMJ products and to exploit all rights, as set out in our licence.

The Submitting Author accepts and understands that any supply made under these terms is made by BMJ to the Submitting Author unless you are acting as an employee on behalf of your employer or a postgraduate student of an affiliated institution which is paying any applicable article publishing charge ("APC") for Open Access articles. Where the Submitting Author wishes to make the Work available on an Open Access basis (and intends to pay the relevant APC), the terms of reuse of such Open Access shall be governed by a Creative Commons licence – details of these licences and which Creative Commons licence will apply to this Work are set out in our licence referred to above.

Other than as permitted in any relevant BMJ Author's Self Archiving Policies, I confirm this Work has not been accepted for publication elsewhere, is not being considered for publication elsewhere and does not duplicate material already published. I confirm all authors consent to publication of this Work and authorise the granting of this licence.

For peer review only

Title page

Title: Implementation Research for taking Tobacco Control Policies to scale in India – A realist evaluation study protocol.

Authors: *Pragati Hebbar[1,4], Vivek Dsouza[1], Upendra Bhojani[1], Onno C P van Schayck[2], Giridhara R Babu[3], Gera E. Nagelhout[4,5]

[1]Institute of Public Health (IPH), 3009, II-A Main, 17th Cross, Krishna Rajendra Rd, Banashankari Stage II, Bengaluru, Karnataka 560070.

[2]Department of Family Medicine, Maastricht University (CAPHRI), P. Debyeplein 1, 6229 HA Maastricht, Netherlands.

[3]Indian Institute of Public Health, Public Health Foundation of India (PHFI), Bangalore, Magadi Rd 1st cross, Next to leprosy hospital, SIHFW premises, Bengaluru, Karnataka 560023

[4]Department of Health Promotion, Maastricht University (CAPHRI), P. Debyeplein 1, 6229 HA Maastricht, the Netherlands.

[5] IVO Research Institute, Koningin Julianaplein 10, 2595 AA The Hague, the Netherlands

***Corresponding Author**: Pragati B Hebbar (pragati@iphindia.org)

For peer review only - http://bmjopen.bmj.com/site/about/guidelines.xhtml

Abstract

Introduction: There are ongoing policies and programs to reduce tobacco use and minimize the associated health burden in India. However, there are several challenges in practice leading to different outcomes across Indian states. Inadequate understanding of how national tobacco control policies achieve their results under varied circumstances obstruct the implementation and scaling-up of effective strategies. This study is a realist evaluation using largely qualitative methods to understand the implementation process of India's tobacco control policies. It will do so by evaluating India's Cigarettes and Other Tobacco Products Act (COTPA) and the National Tobacco Control Program (NTCP). The study aims to examine how, why, for whom, and under which circumstances COTPA and NTCP are implemented in India.

Methods and analysis: A realist synthesis on implementation of tobacco control policies in low- and middle-income countries is conducted. This is followed by qualitative data collection and analysis in three Indian states selected based on data from two rounds of the Global Adult Tobacco Survey. The study comprises of three steps: (1) development of initial program theories, (2) testing and refinement of initial program theories, and (3) testing and validation of refined program theories. We will interview policymakers, program managers, and implementers to identify facilitators and barriers of implementation. The purpose is to identify context-specific evidence-based strategies to gain insights into the implementation process of COTPA and NTCP. Further we aim to contribute to tobacco control research by establishing communities of practice to engage with cross-cutting issues

Ethics and dissemination: The Institutional Ethics Committee, at the Institute of Public Health (Bengaluru) has approved the protocol. Written informed consent forms will be obtained from all the participants. Dissemination has been planned for researchers, policy

For peer review only

makers and implementers as well as the public through peer reviewed publications, conference presentation, webinars, social media updates.

PROSPERO registration: CRD42020191541

KEYWORDS: Realist evaluation, Tobacco control, Implementation, low and middle-income countries, Public health, India.

Strengths and limitations of the study

- Realist synthesis to build on published literature from diverse disciplines to theorize about implementation of tobacco control policies in LMICs.
- Realist evaluation on how implementation and scaling up of implementation of tobacco control law works in three diverse sub-national contexts within India
- Regional consultations and communities of practice to test and refine theories
- Secondary data of enforcement and monitoring of tobacco control policies has challenges of limited data reliability, data segregation and comparability over time.
- Primary data collection of interviews and observations might be impacted by COVID19 related restrictions.

For peer review only

Introduction

Tobacco use is one of the major public health threats killing approximately eight million people globally and over 1.3 million adults in India each year.(1) According to the 2016-2017 Global Adult Tobacco Survey, 28.6% (roughly 267 million) of the adult population uses tobacco in India. In 2011, 14.6% of adolescents (over 35 million people) were current users of varied forms of tobacco products.(2) In the same year, the economic burden of tobacco-related diseases in India was INR. 1,04,500 crores (US$ 22.4 billion) which was 1.16% of the country's GDP.(3)

Prior to the WHO Framework Convention on Tobacco Control (FCTC) ratification in 2004, India passed a comprehensive tobacco control law – the Cigarettes and Other Tobacco Products Act (COTPA), 2003 to prohibit (1) smoking in public places, (2) direct and indirect advertisements of tobacco products, (3) sale of tobacco products to minors and within 100 yards of any educational institutions, and (4) sale without specified graphic health warning labels.(4) To support the implementation of this law, the Government of India launched the National Tobacco Control Program (NTCP) in the year 2007-08 to 1) create awareness regarding the harmful effects of tobacco consumption, 2) reduce the production and supply of tobacco products, 3) ensure effective implementation of the provisions under 'The COTPA 2003, 4) help people quit tobacco use, and 5) facilitate implementation strategies for prevention and control of tobacco advocated by the WHO FCTC.(5) Despite a national policy and program, the outcomes in Indian states is varied. Existing social inequities are compounded with contextual factors such as the differences in political and administrative structures, organizational and individual capacities, and the political economy of tobacco production and consumption within the various states.(6)

Complex policies and their implementation

For peer review only

Policy implementation is complex because of a number of interacting actors involved and the environments in which they are designed and implemented (contexts).(7)(8) Several conceptual and theoretical frameworks on understanding policy and its implementation exist across domains like political science and public administration.(9)(10) For instance, Laswell's linear model outlines five stages of public policy processes – agenda setting, formulation, adoption, implementation, and evaluation, where each stage has its own dynamics and presents several challenges and opportunities.(11) In the field of public health, Walt and Gilson developed the policy triangle framework to analyze the linkages between the policy contexts, policy processes and policy contents, and its influence on the health outcomes while bringing into focus the important role of state and non-state actors.(12) Despite the existing frameworks and literature on macro-level policy analysis, there is a dearth of research and theorization on the implementation process, especially in low and middle-income countries (LMICs).

Realist evaluation approach

The theory-driven inquiry is considered promising in its attempt to evaluate complex phenomena because it examines the causal mechanisms by considering the contexts within which the intervention is embedded that facilitates a particular kind of change.(13)(14)(15) Realist evaluation, a form of theory-driven inquiry, is increasingly used to assess health policies and programs, to explain *what* works for *whom* and under *what circumstances* instead of simply 'does it work or not?'(16) The goal of realist evaluation is to make sense of *conditions* in which interventions work (or not) by generating plausible mechanisms. In tobacco control policy implementation, some of these potential conditions are geographic, demographic, economic, religious, and cultural factors rooted in complex socio-political systems that drive or hinder implementation of policies. The study aims to explain how

For peer review only

certain facilitators and barriers shape implementation of COTPA and NTCP in India, which can be used to inform policymakers about best practices. The current research constitutes a doctoral study that is part of a broader enquiry that aims to inform policy and practice through a DBT/ Wellcome Trust early career fellowship grant. The broad research question that is addressed in this study is: How were tobacco control policies implemented in India between 2003-2018? This is subdivided into three questions that will guide the study.

a. Which states performed better (implementation and outcomes), and for whom (which populations) was it better?
b. How do Indian states differ with respect to tobacco control outcomes?
c. Which factors facilitated successes in implementation in some settings? How were these achievements sustained? And what were the barriers?

Methods and analysis

The study is a realist evaluation using an implementation research approach to examine and explain the facilitators and barriers to implementing COTPA and NTCP in India. Realist evaluation is method neutral and increasingly used to evaluate complex interventions. This approach will be used to develop, test, validate, and refine program theories (also known as middle-range theories) that explain how the contexts (C) in which COTPA is implemented influences a set of mechanisms (M) resulting in particular kinds of outcomes (O). These C-M-O configurations will help understand how COTPA and NTCP work in different settings to produce different outcomes. The study uses the Realist And Meta-narrative Evidence Syntheses: Evolving Standards (RAMESES) II reporting standards for realist evaluation to report the empirical results. The overall study design is depicted in Figure 1.

Step 1: Development of Initial Program Theories (IPTs)

For peer review only - http://bmjopen.bmj.com/site/about/guidelines.xhtml

For developing the IPTs, a realist synthesis is undertaken in the phase 1 of the study followed by a realist evaluation in the phase 2. The purpose of the synthesis is to understand the available literature regarding the facilitators and barriers to implementation of tobacco control policies in LMICs. Within the LMIC group the social and political contexts vary widely. This synthesis aims to capture the diversity of the contexts and mechanisms that have worked or failed to generate desirable outcomes in such settings. Databases to be used to find literature include EBSCOHost and Web of Science. Studies from LMICs that describe and elaborate on how and why tobacco control policies are implemented will be considered. Articles are included if (a) published in English and peer-reviewed (b) it describes the facilitators and barriers to tobacco control policy implementation, (c) discusses mechanisms used in implementing tobacco control policies.

Two reviewers independently screen the abstracts and titles, following which an assessment of quality and rigor is performed. The selected papers are then independently reviewed and coded using common definitions. An initial framework was developed by the research team based on their field experiences and understanding of the existing literature. Based on this initial framework, some codes were derived deductively while there was addition to the list inductively based on the data. Due to the study's qualitative nature, multiple reflexive inter-coder discussions are planned after reviewing full texts to minimize the risk of bias during the study. A detailed realist synthesis protocol has been published earlier in the PROSPERO database.(17) The study will also be guided by the implementation experiences of the principal investigator (PH) and that of the research team that comprises members from LMICs and high income countries coming from diverse backgrounds, which will add a layer of richness to the synthesis process. This would conclude the phase one of the study which comprises of the realist synthesis

For peer review only - http://bmjopen.bmj.com/site/about/guidelines.xhtml

Study Setting: Following the realist synthesis for the realist evaluation Indian states and union territories were ranked by examining proxy indicators of tobacco control policy implementation and placed into three broad categories: (1) better-performing states that show an improvement in the implementation between 2003 – 2018, (2) average performing states that offer no significant change in the implementation between 2003 – 2018 and (3) poor-performing states where the implementation has worsened between 2003 – 2018. For this exercise, data from the Global Adult Tobacco Survey (GATS) India rounds 1 (2009-2010) and 2 (2016 – 2017) were assessed. A shortlist of three states in each category was developed and shared with some actors (researchers, policy advocates and implementers/ decision-makers). One state was selected from each of the three categories by the actors and the research team for detailed case studies. The three Indian states selected are Kerala, West Bengal and Arunachal Pradesh each with widely varying contextual factors. At the end of this phase, the detailed IPTs will help identify and explore contexts, mechanisms, and outcomes (C-M-O) configurations of COTPA and NTCP.

Step 2: Testing and refinement of IPTs

The study will employ predominantly qualitative methods with some quantitative data to refine the initial program theories (IPTs) by exploring the contexts, mechanisms, and outcomes, of COTPA and NTCP in the shortlisted three states. It will consist of:

1. In-depth interviews and focus-group discussions (See sample size and strategy)
2. Review and analysis of implementation documents and data based on the enforcement of COTPA sections from government websites and national crime records bureau portal, review mechanism meetings, and COTPA awareness activities to understand and build the case studies.
3. Participant observation during meetings, and site visits, field notes and reflections of the researchers regarding implementation of COTPA-NTCP.

For peer review only

For peer review only - http://bmjopen.bmj.com/site/about/guidelines.xhtml

Sample Size and Strategy: Interviews and focus group discussions with state and district level stakeholders (about 15-20 in-depth interviews in each of the three states to capture implementers perspectives and at least one focus group discussion per state) will be conducted to understand the challenges and opportunities in implementing tobacco control policies. The stakeholders are purposively selected and range from state and district level program staff, bureaucrats, politicians, officers from police, education, finance, transport, municipalities, gram panchayat (local self-government bodies), tourism, non-governmental organizations working on tobacco control, members of the media etc. An index list will be drawn based on stakeholder mapping for each of the three states and later snowballed to reach out to the relevant stakeholders from these index participants. The information sheet and consent will be sought from stakeholders and an interview guide would guide the data collection.

Inclusion criteria for stakeholder interviews and focus group discussions:

- Participants must be designated decision makers/ managers or implementers
- Participants must have worked in the area of tobacco control within the selected states.

Data collection, management, and analysis: The interviews will be telephonic due to the COVID-19 pandemic and if circumstances permit, they will be conducted in-person, using a semi-structured interview guide to elicit the facilitators and barriers to implementing COTPA and NTCP. The probes include the thematic of education, enforcement, intersectoral coordination based on the initial framework developed by the research team. The guide will be adapted to different stakeholder groups (based on preliminary research on their background, role in the implementation process and experience), tested for relevance, and refined. The research team member will contact the stakeholders by email and phone

For peer review only

explaining the goals and objectives of the study and secure appointments. The team member will also explain the informed consent form and send a copy of it via email and the returned signed copies will be securely stored. The interviews will range between 45-60 minutes and be conducted at a preferred time to minimize stakeholders' inconvenience. As a form of member checking, we will conduct at least one focus group discussion in online or face to face format within stakeholder groups/departments with some individuals who had participated in in-depth interviews in each of the three states to understand their knowledge and perception regarding COTPA and NTCP.

Data collected from the interviews and focus groups will be audio-recorded and transcribed in verbatim. The audio recording, transcripts, and detailed notes taken during each interview will be saved in a secure repository. Internal team guides are developed with clear documentation flow and procedures including file naming for data appropriate management. Analysis of the interview transcripts will be cross checked by two researchers to check the quality and accuracy and identify any interpretation errors. The qualitative data will be organized in data management software -- NVIVO software version 12. In NVIVO, the transcripts will be coded into broad themes relevant to the study. Based on a preliminary coding and analysis, the lead researcher and the research team will identify common themes with the help of thematic analysis that will outline mechanisms that are facilitators or barriers to implementing COTPA in India. The analysis is iterative and guided by the realist approach taking into consideration the complexity and contextual specificity in tobacco control policy implementation in India. Interviews from the three states will be compared and contrasted to enable us to identify similarities, differences, and gaps in tobacco control policy implementation in different contexts. Regular reflexive interactions are planned with the lead researchers' mentor and supervisor from the institute for identifying any issues in the conduct of the study and recalibrating when required. This will allow us to sharpen the understanding

For peer review only

of tobacco control policy barriers and facilitators. The output of the data analysis will help in refining our IPTs. The resulting theories will be then validated.

Step 3: Validation of refined PTs

The refined program theories will be validated during two regional consultations with stakeholders in India and maybe further refined based on the inputs from the consultations. Dissemination of the validated PTs is planned at a final national symposium to meet the policy objective of the fellowship. The regional consultations will be used to validate preliminary findings and program theories with stakeholders for feedback to examine whether the analysis is in line with the knowledge, perceptions, and beliefs of the stakeholders. Following these regional consultations, we envisage developing a community of practice of the participants of the regional consultation. The aim of this community of practice is to provide a platform to share learnings, challenges as well as best practices across departments and different Indian states. The national symposium will facilitate the development of a stakeholders' manual which will incorporate a simplified version of the refined program theories – their relevant C-M-O configurations and best practices to scale implementation of COTPA and NTCP in India.

Ethics and dissemination

The Institutional Ethics Committee, at the Institute of Public Health (Bengaluru) has approved the protocol in two parts. Phase 1 of the study (based on secondary data) was exempted from review, and Phase 2 of the study (largely qualitative) submitted on March 26, 2020, was approved on April 28, 2020 (ref: IEC-FR/01/2020). The team will adhere to the ethical considerations during the conduct of the research as proposed in the submission to the institutional ethics committee and provide them an annual update. The findings of the study

For peer review only - http://bmjopen.bmj.com/site/about/guidelines.xhtml

274 will be disseminated using academic and non-academic channels for the scientific
275 community, government and non-government organizations, and the public. This will
276 facilitate dialogue and encourage the uptake and integration of evidence into practice. For the
277 scientific community, the intended research outputs are to publish several journal articles: the
278 realist synthesis of facilitators and barriers of implementation of tobacco control policies in
279 LMICs, the variation in the status of COTPA implementation across Indian states, theories
280 explaining the implementation of COTPA in three Indian states, and the refined theories on
281 COTPA implementation in India. These will be published as open access journal articles in
282 peer-reviewed journals and as an open-access article-based PhD thesis. The findings will also
283 be presented during seminars and journal clubs organized internally at the Institute of Public
284 Health, Bengaluru, and at national and international public health conferences and webinars.

285 For national and sub-national implementers, an implementers' manual/toolkit
286 comprising of site-specific knowledge, experience, and implementer-wisdom on
287 implementation strategies is planned. Data from the findings and analysis during the study
288 will also be translated into technical reports and policy briefs to update the project progress to
289 its partners and funders. For the public, we aim to develop communication materials that
290 translate scientific information into easily understandable resources on tobacco control
291 implementation. This includes social media platforms (Facebook and Twitter), quarterly
292 updates disseminated via the Institute of Public Health Bengaluru's newsletter: IPH connect
293 and issuing relevant press releases and newspaper columns.

294 We organize bi-monthly webinars on tobacco control implementation (called ‘Inside
295 Implementation’ to bring together policymakers, implementers, the public health community,
296 and those working in tobacco control and health to critically engage in dialogue on the issue
297 of tobacco use). Consultations (regional as well as national) to disseminate the interim and
298 final reports on the program theories of implementation of COTPA and NTCP. And an online

For peer review only - http://bmjopen.bmj.com/site/about/guidelines.xhtml

and offline 'communities of practice' mode of engagement that will bring together state and district-level implementers, policy makers, and diverse stakeholders to work on cross-cutting issues of tobacco control and share best practices in strengthening the implementation process of tobacco control in India.

Limitations of the study

Some of the limitations envisaged include the unavailability, inconsistency, low quality, and inaccuracy of state-level enforcement data. Information from different implementing agencies comprises of different data sets, varying figures, different timelines of data collection, addition of new states and union territories based on the political history of India, and the unavailability of disaggregate data (COTPA violations of Sec 4, 5, 6a and 6b, and 7) necessary for the study. Furthermore, the ongoing COVID-19 pandemic has mandated a change in data collection techniques from in-person interviews to an online (telephonic or video call based) format which might impact the assessment of the non-verbal communication of the stakeholders during interviews. Researchers are mindful of this and whenever possible will try to conduct video based or in-person interviews. A final limitation that we want to mention is that the findings from the three Indian states would not be generalizable as it will capture specific data and diversity in terms of implementation outcomes, geography and socio-political contexts of the three selected states. Hence, they would provide a starting point for enquiries in similar settings and have to be interpreted accordingly.

Patient and public involvement

This study protocol describes a realist evaluation study during which participants ranging from various government departments as well as non-government organizations will be

For peer review only

involved. The engagement with these participants will be for understanding the tobacco control landscape in India, data collection, regional consultations for theory refinement and validation. The involvement will be deepened and sustained by developing communities of practice to exchange learnings and co-produce knowledge related to implementation of tobacco control policies in India.

Acknowledgements – The authors would like to thank Dr. Prashanth NS, DBT/ Wellcome Trust India Alliance Intermediate Fellow at the Institute of Public Health, Bengaluru for his support and inputs on realist evaluation.

References:

1. WHO. WHO report on the global tobacco epidemic, 2019: offer help to quit tobacco use: executive summary. World Heal Organ [Internet]. 2019;1–8. Available from: https://www.who.int/teams/health-promotion/tobacco-control/who-report-on-the-global-tobacco-epidemic-2019
2. WHO. India (Ages 13-15) Global Youth Tobacco Survey (GYTS). Fact Sheet. World Heal Organ [Internet]. 2009;63. Available from: http://www.searo.who.int/entity/noncommunicable_diseases/data/ind_gyts_fs_2009.pdf?ua=1
3. John RM,Rout SK,Kumar BR AM. Report on Economic Burdent of Tobacco Related Diseases in India.pdf. 2014. p. 15–8.
4. The G-I. India - COTPA, 2003 - national.pdf. 2003. p. 9.
5. National Tobacco Control Cell, MOHFW G. Operational Guidelines- National Tobacco Control Programme 2015. 2015;1–66. Available from: http://www.who.int/lep/resources/SEAGLP20062.pdf

For peer review only

6. Nazar GP, Chang KCM, Srivastava S, Pearce N, Karan A, Millett C. Impact of India's National Tobacco Control Programme on bidi and cigarette consumption: a difference-in-differences analysis. Tob Control [Internet]. 2020;29(1):103–10. Available from: http://login.ezproxy.ub.unimaas.nl/login?url=https://search.ebscohost.com/login.aspx?direct=true&db=cmedm&AN=30554161&site=ehost-live&scope=site

7. Sterman JD. Learning from evidence in a complex world. Am J Public Health. 2006;96(3):505–14.

8. Westhorp G. Using complexity-consistent theory for evaluating complex systems. Evaluation. 2012;18(4):405–20.

9. Nilsen P. Making sense of implementation theories, models and frameworks. Implement Sci. 2015;10(1):1–13.

10. Peters DH, Adam T, Alonge O, Agyepong IA, Tran N. Implementation research: What it is and how to do it. Br Med J. 2014;48(8):731–6.

11. Sabatier P. Theories of the Policy Process. Theor Policy Process. 2019;4–7.

12. Walt G, Gilson L. Reforming the health sector in developing countries: The central role of policy analysis. Health Policy Plan. 1994;9(4):353–70.

13. Chen HT, Rossi PH. The theory-driven approach to validity. Eval Program Plann. 1987;10(1):95–103.

14. Van Belle SB, Marchal B, Dubourg D, Kegels G. How to develop a theory-driven evaluation design? Lessons learned from an adolescent sexual and reproductive health programme in West Africa. BMC Public Health. 2010;10.

15. Shearn K, Allmark P, Piercy H, Hirst J. Building Realist Program Theory for Large Complex and Messy Interventions. Int J Qual Methods. 2017;16(1):1–11.

16. Pawson R, Tilley N. An Introduction to Scientific Realist Evaluation. In: Evaluation for the 21st Century: A Handbook. 1997. p. 405.

For peer review only

17. Hebbar Pragati, Dsouza V, Bhojani U, Schayck OC van, Babu GR, Nagelhout GE. PROSPERO registration. 2020.

Authors' contributions – PH was responsible for the funding acquisition and project administration. UB and PH were involved in conceptualization and GN helped in strengthening the methodology. VD and PH were involved in data collection, analysis, writing original drafts. Whereas UB, OvS, GB and GN were involved in supervision, reviewing and editing drafts. All authors read and approved the final manuscript.

Funding statement - The study is supported by the DBT/Wellcome Trust India Alliance Early Career Fellowship awarded to Pragati Hebbar [IA/ CPHE/17/1/503338]

Competing interests – None declared

Patient consent for publication – Not required

List of abbreviations:

COTPA – Cigarettes and Other Tobacco Products Act

CMO – Context – Mechanism - Outcome

DBT – Department of Biotechnology

GATS – Global Adult Tobacco Survey

IPT – Initial program theory

LMIC – Low- and middle-income countries

NTCP – National Tobacco Control Program

PT – Program theory

RAMESES - Realist And Meta-narrative Evidence Syntheses: Evolving Standards

WHO – FCTC – World Health Organization Framework Convention on Tobacco Control

For peer review only

Figure title and legend:

Figure title - Figure 1: Study design

Figure legend – Figure 1 explains the overall study design which includes the realist synthesis followed by a realist evaluation to develop, test and refine program theories explaining the implementation of tobacco control policies.

For peer review only

For peer review only - http://bmjopen.bmj.com/site/about/guidelines.xhtml

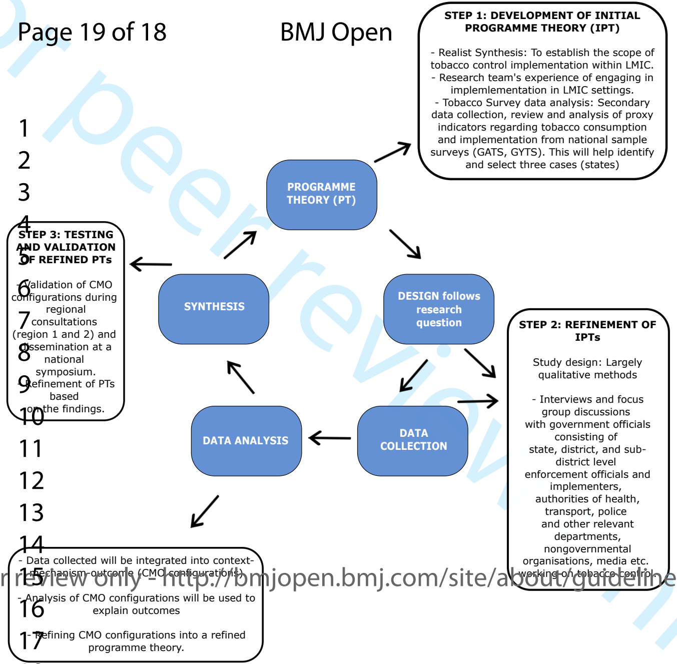

er review only - http://bmjopen.bmj.com/site/about/guideline
