# Supplementary material for: Implementation research for taking tobacco control policies to scale in India: a realist evaluation study protocol
Source: BMJ Open. 2021 May 18;11(5):e050859. doi: 10.1136/bmjopen-2021-050859 (PMC8130733; doi:10.1136/bmjopen-2021-050859)
Supplement: Reviewer comments [file bmjopen-2021-050859.reviewer_comments.pdf]

PEER REVIEW HISTORY

BMJ Open publishes all reviews undertaken for accepted manuscripts. Reviewers are asked to complete a checklist review form (**http://bmjopen.bmj.com/site/about/resources/checklist.pdf**) and are provided with free text boxes to elaborate on their assessment. These free text comments are reproduced below.

ARTICLE DETAILS

**TITLE (PROVISIONAL)**	Implementation Research for taking Tobacco Control Policies to scale in India – A realist evaluation study protocol
**AUTHORS**	Hebbar, Pragati; Dsouza, Vivek; Bhojani, Upendra; van Schayck, Onno; Babu, Giridhara R; Nagelhout, Gera

This article was not externally reviewed at BMJ Open. Protocols that have been independently assessed prior to submission to BMJ Open are usually fast-tracked to publication on the grounds that further substantial changes will not be possible. This independent assessment will usually be external, independent review for both a substantial grant award from a non-commercial or government funder and ethics approval.
